# Application of process analytical technology for real-time monitoring of synthetic co-culture bioprocesses

**DOI:** 10.1007/s00216-025-05949-2

**Published:** 2025-06-07

**Authors:** Nicole A. Dambruin, Jack T. Pronk, Marieke E. Klijn

**Affiliations:** Department of Biotechnology, Delft University of Technology, Van Der Maasweg 9, Delft, 2629 HZ The Netherlands

**Keywords:** Synthetic co-culture, Process analytical technology, Real-time monitoring, Industrial biotechnology, Microbiology, Process control

## Abstract

Synthetic microbial co-cultures can enhance bioprocess performance by division-of-labor strategies that, through spatial segregation of product-pathway modules, circumvent or mitigate negative impacts of the expression of an entire product pathway in a single microorganism. Relative abundance of the microbial partners is a key parameter for the performance of such co-cultures. Population control strategies based on genetic engineering have been explored, but the required interventions may impose an additional metabolic burden and thereby negatively affect co-culture performance. Regulation of co-culture composition by controlled substrate feeding strategies or temperature control requires real-time population monitoring. Process analytical technology (PAT) is an approach for real-time monitoring and control of processes, enabling continuous observation of co-cultivation that may serve as a foundation for population control strategies. In this review, we discuss PAT methods for monitoring synthetic co-cultures, either through direct biomass measurements or by tracking soluble or volatile metabolites. We discuss advantages, limitations, and applications of established as well as emerging technologies and conclude that leveraging PAT for precise, real-time population control has the potential to enhance stability, efficiency, and industrial scalability of synthetic co-cultures.

## Introduction

Microbial biotechnology contributes to the transition towards a sustainable economy by using microorganisms, often subjected to classical strain improvement and/or genetic modification, for the large-scale production of chemicals, pharmaceuticals, and food ingredients from renewable resources [1]. Industrial processes in microbial biotechnology are predominantly based on single microbial strains and operated under aseptic conditions. While simplifying optimization of microorganisms and process parameters, the use of such monocultures also brings about limitations, for example when dealing with complex substrate mixtures and/or dynamic process conditions. Microbial waste-water purification in non-aseptic, open systems demonstrates the power of multi-species microbial communities to address such challenges during the conversion of complex materials and substrates at the end of the anaerobic food chain [2]. However, open mixed communities are difficult to engineer due to complex population dynamics and unknown interactions between the strains [2].

Recently, interest has intensified in exploring the potential of using defined co-cultures of two or more microbial strains for microbial biotechnology applications. Laboratory studies have demonstrated that co-cultivation strategies can help balance product pathways to mitigate byproduct formation and circumvent problems originating from enzyme promiscuity and host-dependent incompatibilities to express specific enzymes [3–5]. Furthermore, removal or production of intermediates by syntrophic species can influence reaction thermodynamics and, thereby, their feasibility [2]. As the species composition of synthetic co-cultures is defined and simpler than that of open mixed cultures, engineering of individual species is possible, as is the case for pure cultures. Despite the potential of synthetic co-cultures for industrial biotechnology, upscaling from laboratory to industrial scale remains a challenge. While open mixed cultures typically have stable chemical, metabolic, and ecological equilibria, those in synthetic co-cultures can be highly sensitive to process parameters. A suboptimal population equilibrium can lead to diminished product titers and yields [6]. To optimize population composition and prevent population instability, monitoring and controlling the relative abundance of the microbial strains in synthetic consortia is a key objective.

The challenge of monitoring the population composition in defined microbial consortia can be addressed by different strategies. One category of strategies involves off-line methods, i.e., methods that require the collection of a sample and analysis on a stand-alone device, sometimes also including sample preparation. Examples to monitor species abundance in co-cultures off-line are rDNA sequencing and PCR analysis [7–9]. Even though off-line methods allow highly precise quantitative and qualitative data, these do not allow real-time monitoring of the population composition. For successful implementation of automated population dynamics control strategies, real-time monitoring of the population composition is required, and off-line analytics are not suitable for this purpose.

Analytical techniques enabling automated monitoring and control are referred to as Process Analytical Technologies (PAT). PAT is defined as an approach for designing, analyzing, and controlling manufacturing processes through timely measurements of critical process parameters [10]. In addition to the traditional pH, dissolved oxygen, temperature, capacitance, and off-gas O_2_ and CO_2_ analysis, measurements of substrates and metabolites using PAT tools can provide deeper insight into the system’s behavior and allow for the development of tailor-made control strategies. Real-time measurements in bioreactors may involve three configurations: (1) in in-line, a PAT sensor is inserted in the process; (2) in on-line, measurements are performed outside the bioreactor using a bypass loop; and (3) in at-line, automated sampling connected to an analyzer. Depending on the PAT tool, a continuous stream of information on biomass composition as well as biomass, substrates, and/or metabolites concentration can be captured, facilitating the opportunity to immediately respond to changing dynamics. The ability to automate and speed up process decisions makes PAT an interesting approach for co-culture population dynamics control.

**Fig. 1:**
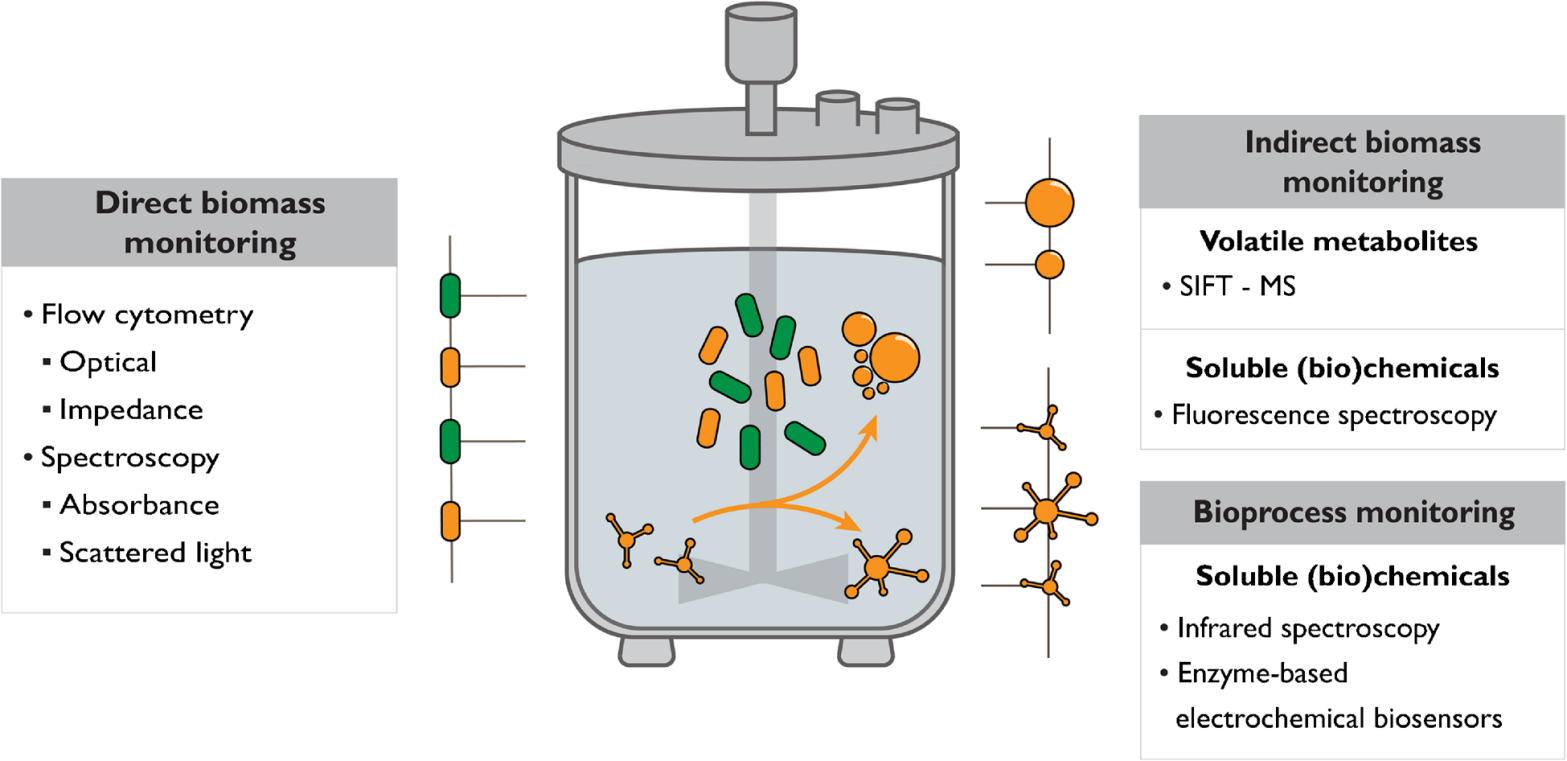
Process analytical technology applied for monitoring of co-cultures per type of analyte. Biomass can be directly measured to assess the biomass composition, or alternatively, soluble and volatile substrates and metabolites from fermentation can serve as proxies for population dynamics in the co-culture. Other co-culture bioprocess parameters were monitored based on soluble (bio)chemical measurements. Abbreviations: SIFT-MS, selected-ion flow-tube mass spectrometry

**Fig. 2:**
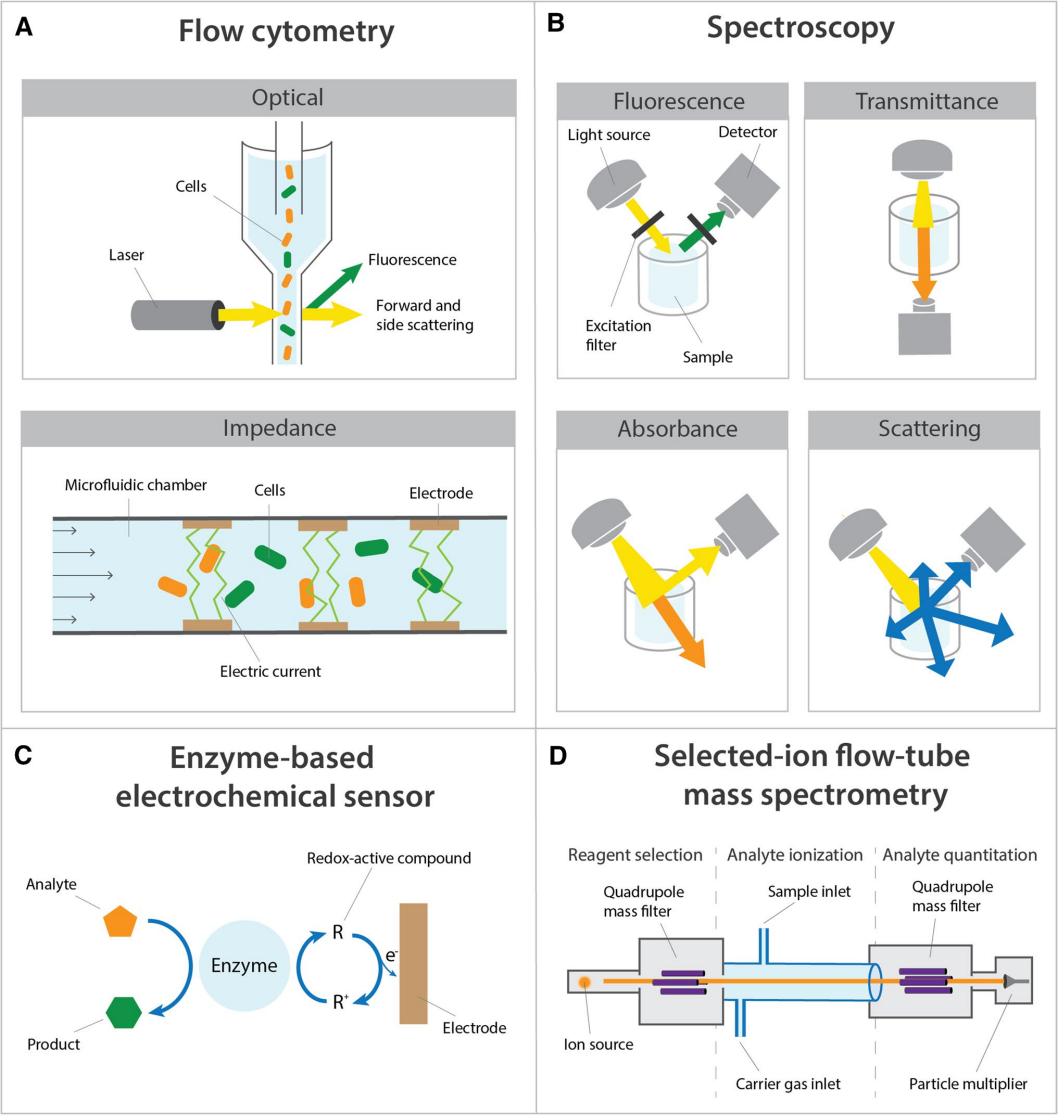
A schematic of the working principle of flow cytometry, spectroscopy, enzyme-based electrochemical sensors, and selected-ion flow-tube mass spectrometry (SIFT-MS). **A** In optical flow cytometry, single cells pass through a laser in a fluid stream. Forward scattering, side scattering, and fluorescence effects can be measured and linked to cell size, cell complexity, and auto-fluorescence. Impedance flow cytometry measures the frequency-dependent polarizability as cells pass through an electric current in a microfluidic chamber. **B** Light-matter interactions employed by spectroscopy, showing fluorescence, transmittance, absorbance, and scattering. **C** In an enzyme-based electrochemical sensor, an enzyme either generates or consumes a redox-active compound (R) during the conversion of a target analyte. The resulting change in the electrical signal is measured and correlated to the concentration of the target substance. **D** SIFT-MS works by introducing a sample into a flow tube where it reacts with precursor ions. The resulting product ions are analysed by a quadrupole mass analyzer to identify and quantify volatile organic compounds in real time

The goal of this review is to describe the current status of research applying in-/on-/at-line PAT for monitoring defined co-culture processes. We focus on PAT that do not involve genetic engineering (e.g., the expression of fluorescent tags). Although techniques that use genetic engineering can give high-resolution optical insight into the abundance of the fluorescent species, the strategy is restricted to microorganisms that are genetically accessible. Moreover, from a bioprocessing perspective, the metabolic burden imposed by the required expression of heterologous proteins, as well as potential mutations in the responsible genes, may negatively affect culture performance under industrial process conditions [11–13]. The first section of this review describes applications of PAT for monitoring interspecies dynamics by using biomass as an analyte. Subsequently, PAT applications are reviewed that measure soluble and volatile (bio)chemicals as a basis for synthetic co-culture monitoring. A schematic overview of the covered PAT applications is presented in Fig. 1. In Table 1, we listed publications that used PAT for monitoring of synthetic co-cultures. Analytical technologies applied to co-cultures covered in this review are summarized in Table 2, including advantages, disadvantages, and case studies reported in the literature. The working principle of the PAT methodologies used for co-culture monitoring is illustrated in Fig. 2. For an overview of off-line monitoring methods or methods involving genetic engineering, we refer to the review of Schlembach et al. [14]. Finally, we discuss how PAT can be implemented in co-culture bioprocesses through feedback control.

## Direct biomass measurements

### Flow cytometry

Flow cytometry (FC) is a technique to detect and measure physical and chemical characteristics of single cells as they pass through a laser in a fluid stream (Fig. 2A). FC generates different readouts, specifically forward scatter (FSC), side scatter (SSC), and fluorescence. FSC is light scattered in the forward direction (i.e., along the same axis as the beam) and is proportional to the diameter of the cell. SSC measures light that is scattered at a 90° angle to the laser beam and is influenced by internal cellular complexity, such as the presence of dense structures, organelles, or a nucleus. Together, FSC and SSC allow for the acquisition of relevant cellular data, such as the number of cells or the size of the cells [15].

FSC and SSC data was used to distinguish between species in an *Escherichia coli*-*Saccharomyces cerevisiae* co-culture [16, 17] and a *Lactobacillus plantarum-Kazachstania bulderi* co-culture [17]. Populations of the yeasts *S. cerevisiae* and *K. bulderi* were gated based on events with FSC signal area higher than 10^5.5^, while those of the bacteria *E. coli* and *L. plantarum* were defined as events with lower FSC values [17]. In both publications, cells were grown in a Segregostat, which is a continuous cultivation set-up originally developed to control phenotypic subpopulations in pure cultures of *E. coli* and *Pseudomonas putida* through substrate pulsing [18]. Martinez et al. first used the Segregostat and FC measurements to control the population dynamics of an *S. cerevisiae*-*E. coli* co-culture [16]. Glucose depletion gave *S. cerevisiae* a fitness advantage over *E. coli*, as yeast can grow on overflow metabolites ethanol and acetate. Upon pulsing a glucose-containing substrate, the specific growth rate of *E. coli* exceeded that of its yeast partner, which enabled continuous control of growth rates and population dynamics. In subsequent work, Martinez et al. developed a model based on metabolic networks to predict the pulsing frequency [17]. The predictive model was successfully applied in a Segregostat to a cooperative co-culture (*K. bulderi* and *L. plantarum*) and a competitive co-culture (*E. coli* and *S. cerevisiae*). In this case, FSC and SSC signals were used to monitor the co-culture in real time to enable the control strategy.

In FC, fluorescent channels can be used to detect and quantify fluorescence in a specific range of wavelengths, thereby characterizing a distinct cell population. Distinguishing subpopulations by FC can be performed by expressing one or more heterologous fluorescent proteins to mark (an) individual strain(s) [11, 19–21]. Alternatively, at-line biomarker staining (e.g., DNA, RNA, or lipids) for gram-negative bacteria with purpose-built automated staining devices has been successfully applied to circumvent the metabolic burden of heterologous fluorescent protein expression [22, 23]. In aquatic ecosystems with undefined microbial communities, staining with SYBR® Green I and subsequent FC could discriminate individual prokaryotes based on nucleic acid content of bacterial cells [23, 24]. Yet, discrimination between individual prokaryotes with biomarker staining, with the goal to enable real-time co-culture monitoring, faced complications. For example, phenotypic changes resulting from co-cultivation of bacteria can have a profound effect on cell concentration estimates by FC analyses on SYBR® Green I-stained bacteria [25]. These effects originated from interactions between the bacterial co-cultivation partners, since similar FC analyses on mock communities, constructed by mixing different biomarker-stained axenic cultures, outperformed strain-specific qPCR and 16S rRNA gene amplicon sequencing in terms of species quantification. In another study, Haberkorn et al. attempted to quantify the abundance of individual prokaryotic species (*Tistrella mobilis*, *Pseudomonas pseudoalcaligenes*, and *Sphingopyxis*) in a defined co-culture with phototrophic *Chlorella vulgaris* by FC with at-line biomarker staining [26]. Emissions collected on the F11/FL3 fluorescent channels measured the stained nucleic acids of the bacterial cells and chlorophyll autofluorescence of the algal cells, thereby allowing for automated monitoring of the population sizes of bacteria and algae. However, this approach could not discriminate the prokaryotic subpopulation because co-cultivation of algae and bacteria caused a shift of the localization of the prokaryotes on the fluorescent channels. Therefore, monitoring of the prokaryotic populations following the methods as described by [24] was not possible.

### In situ microscopy

Off-line characterization of microbial species by microscopy is a widely established method. Over the last decades, in situ optical microscopy and deep learning image analysis algorithms enabled rapid extraction of quantitative information on microbial cells from microscopy data. Examples include monitoring of fungi [27], microalgae [28, 29], mammalian cells [30–32], and yeast cells [33, 34] with in situ microscopes to determine growth and, in the case of algae, intracellular fatty acid accumulation. Although in situ microscopy is not yet applied to monitoring of synthetic co-cultures, Gustavsson et al. successfully applied this method to detect and quantify fungal contaminations (by the yeasts *Candida utilis* or *Pichia stipitis*) in hybridoma cell cultures [35]. Furthermore, unicellular planktonic species could be identified from water samples using an in situ imaging system and convolutional neural networks [36]. This shows that in situ microscopy can be used for species identification using custom-made image processing algorithms.

Imaging FC is a microscopy technique that combines the high-throughput sampling of traditional FC with individual cell imaging, thereby enabling direct visualization of cellular properties. In addition to cell area, also more complicated metrics can be assessed to provide information on marker localization and cell morphology (e.g., shape, biomarker intensity, texture, and granularity) [37]. Studies focusing on morphological characteristics and spatial distributions have employed imaging FC combined with staining agents to analyse the lipid content of algal cells [38], cell-cycle phase in yeast [39], and 3D structures of mammalian cells [40]. Because of the high specificity of imaging FC and its ability to detect multiple biomarkers, it has the potential to quantify morphologically similar species that cannot be distinguished by only using FSC and SSC.

Co-cultures of species with significantly different cell sizes can be challenging to monitor using microscopy-based techniques because of the required magnification. Guez et al. used a 40 × objective for hybridoma cells [32], the same as for yeast [34], whereas a 10 × objective was used for the algae *Chlamydomonas reinhardtii* [29] and the yeast *Pichia pastoris* [27]. Gustavsson et al. reported that *E. coli* cells present as contaminants in a hybridoma cell culture could not be depicted as separate objects, even at the highest (non-specified) magnification [35]. The authors suggested that high-resolution in situ microscopy could resolve this issue. Alternatively, specific staining can be used to address this problem. For example, Schiavone et al. monitored adhesion events of bacteria and yeast with imaging FC (60 × magnification) by tracking *E. coli* cells with heterologously expressed GFP [41]. Based on currently available information, the FSC signal of FC may be more suitable for quantifying co-cultures of species with large size differences compared to imaging FC because of its relative simplicity, lower costs, and availability in many life-science laboratories.

**Table 1:** Overview of publications using process analytical technology (PAT) for monitoring of synthetic co-cultures

PAT	Organisms	Analyte	Analysis mode	Cultivation mode	Applied data analytics	Control applied?	Reference	Notes
Flow cytometry	*Tistrella mobilis*, *Pseudomonas pseudocalcaligenes* and *Sphingopyxis* sp.	Biomass	At-line	35 mL working volume, 100 mL Erlenmeyer flask	FC data treatment algorithm (*flowCore* (v1.382) and *Phenoflow* (v1.1.2) packages in R)	No	[26]	Used SYBR Green I for staining of nucleic acid
Flow cytometry	*Escherichia coli* and* Saccharomyces cerevisiae*	Biomass	At-line	1 L working volume, stirred bioreactor (Segregostat)	FC data treatment algorithm (MiPI Flow Cytometry Analysis toolbox)	Yes	[16]	Gating based on FSC signal
Flow cytometry	*Escherichia coli* and *Saccharomyces cerevisiae; Lactobacillus plantarum* and* Kazachstania bulderi*	Biomass	At-line	1 L working volume, stirred bioreactor (Segregostat)	FC data treatment algorithm (MiPI Flow Cytometry Analysis toolbox)	Yes	[17]	Gating based on FSC signal
Impedance flow cytometry	*Bacteria (bacilli*, *cocci*, and *vibrio)* and bacilli (*Escherichia coli* and *Salmonella enteritidis)*	Biomass	At-line	Not cultured in co-culture, colonies from plate were diluted in 10 mM PBS buffer solution	Convolutional neural network to analyse impedance data and differentiate bacteria	No	[42]	
Scattered light spectroscopy	*Kluyveromyces marxianus* and *Lactococcus lactis*	Biomass	In-line	Microtiter plate	Partial least square from individual biomass concentrations	No	[43]	
UV/Vis absorbance	*Methylomicrobium buryatense* and *Scheffersomyces stipitis*	Biomass	In-line	Volume not specified	Partial least square from individual biomass concentrations	No	[44]	OD wavelength 269–1100 nm
Fluorescence spectroscopy and UV/Vis absorbance	*Pseudomonas putida* and* Escherichia coli*	Biomass and pyoverdine (metabolite of *P. putida*)	In-line	20-mL bioreactor	Coupling absolute OD value, time derivative of OD, absolute fluorescence value and time derivative of fluorescence to individual biomass concentrations	Yes	[45]	Absorbance measured at 600 nm
Infrared spectroscopy	*Streptococcus thermophilus* and* Lactobacillus bulgaricus*	Viscosity of milk	In-line	60-mL glass bottles in water bath	Principal component analysis relating rheological data and conventional quality parameter values	No	[46]	
Enzyme-based electrochemical biosensor	*Acetobacterium woodii* and *Clostridium drakei*	Lactate	On-line	1.4 L working volume, stirred bioreactor	-	Yes	[47]	
Selected-ion flow-tube mass spectrometry	*Serratia rubidaea*, *Serratia marcescens*, and *Escherichia coli*	Ammonia, ethanol, acetaldehyde, propanol, acetoin, acetone, and acetic acid	At-line	15-mL vials	Principal component analysis	No	[48]

**Table 2:** Process analytical technology (PAT) used in literature for species identification or synthetic co-culture monitoring with the respective analytes, advantages, disadvantages, and data analysis tools

Technique	Analyte	Advantages	Disadvantages	Data analysis tool	Ref
Optical flow cytometry	Biomass	Established method, ability to directly measure biomass based on size and/or morphology	Challenging to distinguish cells with similar size and morphology	FC data treatment algorithm (e.g., specialized software like *FlowJo* or open-source tools like *FlowCore* (R) or *FlowKit* (Python))	[26, 28, 49]
Impedance flow cytometry	Biomass	Ability to directly measure biomass based on capacitance differences of the cell	Not established for co-culture control, sensitive to medium variation	MATLAB tools (not specified), optional: (convolutional) neural network for microbe identification	[42, 50, 51]
Infrared spectroscopy	Substrates, metabolites	Technically simple to implement (in-line), established method for chemical compound monitoring	Interference of water in spectra, only applied to monitoring of matrix	Multivariate data analysis, e.g., partial least square or principal component analysis, relating spectra to analyte concentration	[46, 52]
Fluorescence spectroscopy	Biomass, substrates, metabolites	Technically simple to implement (in-line), established method for fluorescent chemical compound monitoring	Interference from fluorescent medium compounds when measuring biomass, limited to fluorescent analytes	Multivariate data analysis, e.g., partial least square or principal component analysis, relating spectra to analyte concentration	[53–55]
Scattered light spectroscopy	Biomass, substrates, metabolites	Extended linearity compared to absorbance measurements	All particulate scattering is captured, only established in microtiter plates	Multivariate data analysis, e.g., partial least square or principal component analysis, relating spectra to analyte concentration	[43, 56, 57]
UV/Vis absorbance	Biomass	Established method (especially for total biomass concentration measurements)	Limited sensitivity for individual biomass measurements	Lambert–Beer for total biomass concentration, partial least square or principal component analysis relating spectra with analyte concentration	[44, 58]
Enzyme-based electrochemical biosensors	Substrates, metabolites	Sensitive, easy to implement, (complicated) data analysis is not required	Limited to measuring one analyte per sensor	-	[47, 59]
Selected-ion flow-tube mass spectrometry	Volatile organic metabolites	Ability to measure volatile compounds	Variable environmental factors may affect results, limited to volatile organic analytes	Multivariate data analysis, e.g., partial least square or principal component analysis relating spectra to analyte concentration	[48, 60]

### Impedance spectroscopy

Biomass can also be determined based on the frequency-dependent polarizability of cells as a response to an alternating electric field. This method is known under various names, including impedance spectroscopy, dielectric spectroscopy, capacitance measurement, and permittivity measurement. Cell polarizability depends on different variables related to the cell state, described by the Cole–Cole equation [61, 62]. Parameters of the Cole–Cole equation allow for the derivation of additional information about cells, such as their size [63] and specific growth rate [64] as well as their responses to nutritional status [65] and chemical stress factors [66]. Although dielectric spectroscopy was used to monitor (among others) cell size, shape, concentration, and growth phases for different species during growth in pure cultures [67], we have not found studies in which this technique was applied to synthetic co-cultures.

Over the last two decades, high-throughput analysis of single-cell dielectric properties was established using impedance FC. Using this technique, at-line measurements were performed on single cells (~ 1000 cells/s) in microfluidic systems to measure polarizability, membrane capacitance, and cytoplasm conductivity, thereby allowing cell characterization (Fig. 2A). Zhang et al. distinguished three groups of bacteria with different morphologies (bacilli, cocci, and vibrio) and two species of bacilli (*E. coli* and *Salmonella enteritidis*) using impedance FC in combination with deep learning approaches [42]. Impedance FC was also applied to distinguish different mammalian cell types, such as various tumor cell lines, red blood cells, and red cell ghosts [68, 69]. A challenge of impedance FC is the sensitivity to variation in medium conductivity, which can lead to inaccurate results when medium composition changes throughout a fermentation process [51]. Based on the currently available results, impedance FC shows potential to monitor and control synthetic co-cultures in a similar fashion as optical FC but based on cells’ conductivity. However, further development is needed to improve user-friendliness, reliability, and cost-effectivity of impedance FC systems [50].

### Fluorescence spectroscopy

In fluorescence spectroscopy, light excites a fluorophore to a higher energy state, and a photon is emitted at a different wavelength as the molecule relaxes to the lower energy state (Fig. 2B). The range of frequencies (i.e., energy) of emitted photons as a result of a particular excitation frequency is known as the emission spectrum. Constructing a matrix consisting of the emission spectra for a range of excitation frequencies can be used to collect information on all fluorescent compounds in a sample. Emission spectra of the amino acids tryptophan, phenylalanine, and tyrosine function as a unique cellular autofluorescence fingerprint. This fingerprint was utilized off-line to differentiate between lactic acid bacteria and yeast, between different yeast species, and between different bacterial species [53, 54]. Nevertheless, the application of an autofluorescence signal for real-time biomass quantification is not straightforward, as it requires signal calibration for different organisms and growth media. In both studies, cells had to be suspended in distilled water or saline solutions to avoid fluorescence interference of the media components with the low-intensity autofluorescence of cells. This is different for organisms with distinct autofluorescent molecules, such as photosynthetic pigments. Using a portable fluorescent sensor platform, green algae (*Chlorella vulgaris*) and cyanobacteria (*Spirulina*) were quantified by measuring chlorophyll *a* and phycocyanin fluorescence emission spectra, respectively. Biomass could be classified and quantified from pre-mixed cultures of the two species with a 2–16% error in biomass concentration prediction, which reflected an absolute deviation of 4–39 mg/L over a range up to 250 mg/L [55].

A new development where the autofluorescence fingerprint is combined with FC is called spectral FC. In classic FC, excitation with one specific wavelength is followed by the collection of one emission peak at a given wavelength. Spectral FC controls only the excitation and collects an emission spectrum across many wavelengths, similar to fluorescence spectroscopy [70]. The increased number of wavelengths detected in spectral FC allows for the identification of specific fluorochromes or a spectral fingerprint of the cell. Spectral FC has been used in clinical contexts for (off-line) monitoring of therapeutic responses [71] and immune cell characterization [72]. We have not encountered examples of studies in which spectral FC was applied for species identification. Given the potential of autofluorescence-based cell characterization and FC, studies on the potential of this technique for monitoring population dynamics in synthetic co-cultures appear to be highly relevant.

### Scattered light spectroscopy

When a laser light that passes through a sample, particles or molecules scatter the light in all directions (Fig. 2B). The intensity and wavelength of the scattered light depend on the size, shape, and composition of the particles, as well as the wavelength of the incident light. The scattered light can be measured across different positions over a wide excitation spectrum. The resulting spectra depend on the composition of the cytoplasm, cell wall characteristics, cell shape, and cell size [56, 57]. Using a fluorimeter system, Geinitz et al. demonstrated in-line quantification of the abundancies of the bacterium *Lactococcus lactis* and the yeast *Kluyveromyces marxianus* in microtiter-plate co-cultures based on scattered light intensity [43]. Spectral differences were mainly attributed to intracellular compounds and directly corresponded to the biomass concentration rather than to produced metabolites. Despite an overlap of scattered light spectra, multivariate data analysis allowed for differentiation between the species. Up to 4 g/L off-set cell dry weight within a 12 g/L range compared to the reference off-line method was reported. Assessment of the accuracy of this method was difficult because both the in-line and off-line measurements were influenced by changing cell size. Moreover, since the study was performed in microtiter plates [43], experiments with light scattering techniques in a bioreactor context are required to obtain more insight into signal interference by other particles, such as gas bubbles.

### UV/Vis absorbance

Absorbance, also referred to as optical density, measures the amount of light absorbed by a sample at a specific wavelength (Fig. 2B). Based on the Lambert–Beer law, absorbance can be used to determine the concentration of a substance in solution. Wavelengths in the range of UV and visible light regions are often employed for absorbance measurements. Off-line absorbance measurements on microbial communities have been based on pigments (especially chlorophyll *a* and *b*), which occur in phototrophic microorganisms and show absorbance peaks at different wavelengths. This allowed quantification of different types of phototrophs in co-cultures of coccoid cyanobacteria and brown algae (*Cyclotella* sp.) and in co-cultures of *Nannochloropsis salina* and *Phaeodactylum tricornutum*, with prediction errors between 0.4 and 13.4% [58]. Furthermore, Stone et al. calibrated absorbance measurements to determine individual cell concentrations in a co-culture of the bacterium *Methylomicrobium buryatense* and the yeast *Sch. stipitis*, with an average percentage error of up to 5% for individual species biomass concentrations up to 1.75 g/L [44]. It should be noted that this method was only applied in an off-line manner, as samples were centrifuged and diluted in water prior to absorbance measurements. Conversion of this methodology to at-line measurements should be achievable, as automatic dilution of the samples is required to guarantee that high biomass concentrations fall within the linear range of detection.

### Infrared and Raman spectroscopy

Infrared (IR) spectroscopy measures the light that is absorbed at wavelengths in the IR region, which corresponds to specific vibrational state transitions of molecules in the sample [52]. Each molecule produces a characteristic absorption pattern, or “fingerprint,” based on its functional groups and structure. The outputs are spectra that reflect the chemical composition and abundance of the molecules in the sample. An important drawback of IR spectroscopy is the interference of water molecules, which can overshadow peaks belonging to (bio)chemical compounds of interest. Raman spectroscopy, on the other hand, is based on light scattering rather than absorbance and is considered complementary to IR spectroscopy. Raman spectroscopy measures inelastic scattering from a monochromatic radiation source (e.g., lasers) [73], where the resulting spectra are the sum of the scattering effects caused by all Raman-active components in a sample. Raman-active compounds exhibit a change in polarizability during molecular vibrations. This provides a unique spectral fingerprint of the sample’s chemical composition. Although Raman spectra are not subject to large interference from polar molecules such as water, fluorescent molecules can cause interference [74].

In-line Raman and IR spectroscopy are popular PAT tools because of their rapid and non-invasive nature [75, 76]. Yet, only one publication reports on its applicability for analysing microbial co-cultures. Grassi et al. used IR spectroscopy to monitor lactic acid fermentation of milk by a co-culture of *Streptococcus thermophilus* and *Lactobacillus bulgaricus* [46]. By combining transmission and reflectance principles depending on the viscosity of the milk, the authors followed the milk’s texture in-line and in real time. Curd development caused by lactic-acid induced protein denaturation described by the IR data could be associated with pH, acidity, and lactic acid concentration, but correlation to co-culture population composition was not assessed. Other studies suggest that high biomass concentrations in monocultures cause a baseline shift in the in-line Raman and IR spectra due to particulate scattering effects rather than molecular scattering or light absorption [77, 78]. Moreover, available analytical models for biomass determination in monocultures often do not address the origin of observed peaks (e.g., [79, 80]), and it is therefore unclear if spectral peaks correspond with intracellular compounds. This complicates direct discrimination of specific species. Nevertheless, successful analyses of lipid content in algae and carotenoid content in yeast monocultures using in-/on-line Raman spectroscopy show its potential to monitor intracellular compounds in real time [81, 82]. Other applications include cell sorting devices coupled to Raman spectroscopy (Raman-activated cell-sorting [83]). Single-cell Raman spectra can reflect phenotypic and intrinsic biochemical fingerprints of cells and have been used to detect and sort microbes from mixed cultures. For example, unculturable carotenoid-containing bacteria could be discovered from Red Sea samples and lipid-rich *Rhodotorula glutinis* could be sorted after mixing with an *S. cerevisiae* cell suspension [84, 85]. A current challenge of this technique is the low throughput due to the small cross section of spontaneous Raman scattering. Research to overcome this limitation is in progress, resulting in a current maximum throughput of ~ 13 cells per second [85].

## Indirect biomass monitoring

### Soluble (bio)chemicals

Quantitative and direct biomass measurements of co-cultivated species or strains with similar morphologies are complex tasks considering the characteristics of the available PAT. When direct biomass measurements are not possible, real-time measurements of the species-specific metabolites offer an alternative way to estimate biomass concentrations via empirical or model-based correlations. We identified two studies that applied this approach to synthetic co-cultures.

Lee et al. quantitatively monitored *P. putida* and *E. coli* in co-culture using a combination of absolute optical density and fluorescence spectroscopy as well as a time derivative of the optical density and fluorescence spectrum [45]. Abundance of *P. putida* correlated well with the fluorescent signal, which was attributed to the *P. putida* extracellular metabolite pyoverdine. This metabolite showed a signal at a wavelength at which *E. coli* cultures showed negligible fluorescence. As pyoverdine production by *P. putida* was variable depending on temperature, data-driven mathematical models were required for the quantification of *P. putida*. In 7-day turbidostat runs, Lee et al. reached a consistent 10% offset between the on-line estimated composition and the flow cytometric ground truth, possibly caused by biofilm formation [45].

The second metabolite-based study on population composition in synthetic co-cultures employed a biosensor [47]. In the reported batch co-culture, *Acetobacterium woodii* produced lactate from H_2_ and CO_2_. Subsequently, the lactate was consumed by *Clostridium drakei* to produce caproate. The lactate concentration could be measured on-line with enzyme-based electrochemical biosensors and was used to control the H_2_ feed. Enzyme-based electrochemical biosensors are highly sensitive, and their outputs do not require complicated data processing and analysis (Fig. 2C). However, enzymes can be sensitive to harsh environments, resulting in lower reproducibility [59]. While the study of Herzog and coauthors did not directly use the lactate concentration to determine population dynamics [47], it did allow for matching of the lactate production rate of *A. woodii* to the lactate consumption capacity of *C. drakei*. This example demonstrates the potential of real-time control to optimize substrate feeding for synthetic co-cultures.

### Volatile (bio)chemicals

Volatile compounds generated by microbial metabolism can serve as a basis for monitoring synthetic co-cultures via off-gas analysis. Organic volatile compounds (VOCs) are of special interest, as these are often tightly connected with specific metabolic pathways and can be quantified at-line with spectrometric techniques, such as selected-ion-flow-tube mass spectrometry (SIFT-MS). SIFT-MS uses microwave plasma to ionize air and water vapor to form positive and negative precursor ions that are separated into single ionic species in a quadrupole mass filter and injected into a carrier gas (Fig. 2D). This carrier gas flow is then introduced into a flow tube where the ions interact with the VOCs in the sample. A second mass spectrometer (usually a quadrupole) analyzes the product ions based on charge and mass. Most research related to biomarker VOCs aims to identify specific microorganisms through off-gas analysis, as, for example, applied to human breath [86]. Only a few papers explored the application of VOC analysis to study population dynamics or control strategies in microbial co-cultures. Sovová et al*.* monitored the population dynamics of a batch co-culture consisting of *Serratia rubidaea*, *Serratia marcescens*, and *E. coli* by measuring ammonia, ethanol, acetaldehyde, propanol, acetoin, acetone, and acetic acid in the gas phase with SIFT-MS [48]. This work showed that, even in binary mixtures of species of the same genus, the headspace gas composition can function as a fingerprint for specific bacterial species. However, because an orthogonal method to determine the culture composition in the co-cultures was not reported, quantification accuracies are unknown. In addition, the publication mentions “rapid analysis” of the volatile compounds, but the exact duration of the measurements was not specified. Other work shows how the population dynamics of *Salmonella aureus* and *E. coli* were reflected by the profiles of 3-methylbutanoic acid and 3-methylbutanal, which are characteristic VOCs for *S. aureus*, and of indole, a characteristic marker of *E. coli* [60]. Although these measurements were not performed in real time, the reported work does show the potential of VOC profiling for real-time measurements. Further exploration of this approach should include how environmental factors such as temperature, pressure, and humidity in fermentation set-ups affect the performance of MS-based techniques.

For both soluble and volatile (bio)chemical-based approaches, it should be noted that the use of metabolite concentrations to infer population composition implicitly assumes a consistent correlation of metabolite production with the abundance of the responsible microbial species or strain. This assumption may not always be valid due to varying metabolic states, substrate availability, or inter-strain interactions [87, 88]. Therefore, data analysis models must be extensively calibrated with representative data, if necessary, under different regimes, to correlate metabolite concentrations to biomass concentrations.

### PAT for co-culture control

The studies covered in this review focus on assessing the suitability of various PAT tools for co-culture monitoring, often with the primary goal to generate data with higher temporal resolution or to resolve co-culture population dynamics. Only a few papers report on more advanced applications of real-time data, such as process control. In feedback-based process control, the control variable is continuously measured and compared against a predefined target. For instance, in pH regulation, a pH probe monitors the pH level, which is then compared to a set target. If a deviation from the setpoint occurs, the controller activates adjustments to restore balance. In the case of pH control, a base is titrated into the broth to correct the pH value. To regulate population dynamics in co-cultures, researchers have investigated various control strategies. Some approaches involve genetic engineering, such as incorporating intracellular signaling mechanisms (e.g., quorum sensing circuits [89]) coupled to lysis switches [12], or enabling growth through gene expression (e.g., antibiotic resistance cassettes or anti-toxins) [11, 90, 91]. Other control strategies exploit differences in optimal growth conditions under varying environmental factors. These include adjusting culture pH [92], temperature [93], medium supply (e.g., glucose or nitrogen source) [16, 94], or controlling the supply of essential amino acid to auxotrophs [95, 96].

Controllers prevalent in the bioprocessing field include simple ON–OFF control, where the controller is switched on when the error is positive and switches off when the error is zero or negative. Alternatively, the proportional-integral-derivative (PID) controller, a classic control algorithm commonly implemented across engineering, is applied in multiple papers discussed in this review [45, 47]. PID controllers apply three control terms: (1) proportional control (“P” term), which reacts to current error; (2) integral control (“I” term), which addresses accumulated past errors; and (3) derivative control (“D” term), which predicts future errors. PID controllers are computationally simple to implement and do not require a model or deep understanding of the biological system for tuning [97]. Lee et al. reported that population composition in a 7-day turbidostat experiment could be stabilized by integrating a PI control algorithm to tune population dynamics based on temperature changes [45]. The species in the co-culture (*E. coli* and *P. putida*) responded with different time scales to changing temperatures, which was used to further optimize the control strategy. Next to optimizing population dynamics in co-cultures, process yield can be optimized by controlling other variables, such as accumulation of metabolic intermediates. Herzog et al. prevented accumulation of the transfer metabolite lactate using a PID control-based H_2_ feed [47]. Real-time measurements minimized substrate addition and the results of this study provided insight into the fluctuating lactate uptake rate throughout different phases of the fermentation.

Advanced control strategies can be applied when real-time data acts as input for model predictive control, which integrates measurement data with knowledge about metabolism and interspecies interactions. Model predictive control uses a dynamic model of the system to predict future behavior and optimizes control actions over a time horizon. At each step, the algorithm solves an optimization problem to find the best control inputs that minimize a cost function while satisfying constraints. Martinez et al. implemented such a model predictive control strategy, where interactions and long-term effects of substrate pulsing were predicted based on knowledge about metabolic networks and temporal culture composition measured by real-time FC [17]. This study illustrates that a combination of mechanistic modelling and real-time data has the potential to predict the course of the fermentation in real time.

Model predictive control based on mechanistic models relies on a deep understanding of species and processes to effectively utilize limited real-time data (e.g., lactate concentration or FC data) for decision-making. In cases where such detailed knowledge is not available, data-driven modelling can accelerate process control implementation for synthetic co-cultures without requiring extensive prior knowledge. Integrating multiple PAT sensors (e.g., monitoring biomass, soluble and volatile biochemical compounds) alongside standard process parameters (pH, temperature, dissolved oxygen) provides a black-box process understanding. This approach can serve as a foundation for data-driven models, as previously demonstrated for monocultures [98]. However, a purely data-driven approach risks oversimplification, potentially leading to unreliable correlations. A hybrid approach that integrates mechanistic and data-driven modelling enables real-time model refinement and improves the interpretation of unexpected events [99]. Regardless of the chosen modelling strategy, real-time PAT integration for co-culture bioprocesses will be essential for population dynamics monitoring and predictive control.

### Outlook

Synthetic microbial co-cultures have the potential to expand the solution space for industrial biotechnology by several advantages, including division of labor and mitigation of byproduct formation [2]. In many co-culture processes, control of population dynamics is required to optimize production titres and maintain a stable community composition. PAT can play a key role in the development of control strategies by providing real-time process data and facilitate direct actuation when performance parameters are not met. Monitoring co-culture population dynamics requires real-time identification and quantification of species, and ideally information about the phenotypic state of the cells. These parameters can be measured through direct biomass measurements or based on soluble or volatile (bio)chemical concentrations. As the selection of a suitable analytical technique depends on species-specific traits, including but not limited to intracellular carotenoids, cell wall structures, or specific metabolites, it is considered impossible to identify a single PAT tool that is applicable to all co-cultures.

FC is an established PAT for co-culture biomass monitoring based on cell size, internal complexity, and autofluorescence. However, FC cannot differentiate between morphologically similar populations based on FSC and SSC signals. This limits the application of classic FC for the analysis of synthetic co-cultures composed of different engineered strains of the same species [100]. Moreover, variations in phenotypic states, such as cell aggregate formation or varying cell size, can strongly influence the accuracy of the FC measurements [25]. To overcome the limitations of using solely FSC and SSC signals, many promising developments are reported where FC is integrated with other techniques, such as microscopy and spectroscopy (impedance, fluorescence, and Raman). Microscopic techniques can provide spatial information, whereas spectral techniques provide cell-specific (bio)chemical information. The combination with the fluidic systems in FC equipment results in high-throughput single-cell measurements that analyze cells based on characteristics complementary to classic FC output. Nevertheless, more research is needed to explore applications in co-culture bioprocesses, specifically in terms of distinguishing different and similar species, the influence of medium compositions, measurement times in relation to required control responses, and data analytical workflows. In addition, FC is a sophisticated technique that requires specialized operators and a significant investment. Developments to make advanced FC techniques more affordable and accessible to research and development labs can play a key role in unlocking their potential for co-culture bioprocess control.

In addition to combining analytical techniques into one device, it is also envisioned that combining separate PAT for soluble and volatile (bio)chemical detection in a single setup will provide a holistic view on process performance and co-culture metabolism. In turn, this may provide data to build more representative metabolic models that lie at the core of advanced model predictive control strategies. To achieve an integrated approach to quantitatively capture soluble and volatile analytes, a key focus area is gas phase PAT. Currently, off-gas monitoring of co-cultures consists of the application of SIFT-MS, while volatile measurements in monoculture bioprocessing also apply techniques such as chemical sensors [101, 102], gas chromatography [103, 104], or other mass spectrometry-based techniques [105]. This highlights the wide array of techniques to be explored for co-culture specific volatile biomarkers and to assess their potential for population dynamics control.

The application of co-cultures in biotechnology is still developing, especially in terms of operational simplicity, costs, and versatility of different organisms. To optimize co-culture bioprocesses and realize its industrial potential, PAT is essential for generating data that builds performance understanding and enables effective control. The unique characteristics of population dynamics and varying population compositions require co-culture-specific analytical target profiles. These profiles will guide the PAT field to extend the application of existing analytical techniques as well as the development of novel sensors, thereby providing a wide range of tools for co-culture-specific bioprocess control strategies.
